# Evolution of virulence in opportunistic pathogens: generalism, plasticity, and control

**DOI:** 10.1016/j.tim.2012.04.005

**Published:** 2012-07

**Authors:** Sam P. Brown, Daniel M. Cornforth, Nicole Mideo

**Affiliations:** Centre for Immunity, Infection, and Evolution, School of Biological Sciences, University of Edinburgh, West Mains Road, Edinburgh EH9 3JT, UK

**Keywords:** virulence, pathogenicity, evolution, plasticity, regulation, antivirulence drugs.

## Abstract

Standard virulence evolution theory assumes that virulence factors are maintained because they aid parasitic exploitation, increasing growth within and/or transmission between hosts. An increasing number of studies now demonstrate that many opportunistic pathogens (OPs) do not conform to these assumptions, with virulence factors maintained instead because of advantages in non-parasitic contexts. Here we review virulence evolution theory in the context of OPs and highlight the importance of incorporating environments outside a focal virulence site. We illustrate that virulence selection is constrained by correlations between these external and focal settings and pinpoint drivers of key environmental correlations, with a focus on generalist strategies and phenotypic plasticity. We end with a summary of key theoretical and empirical challenges to be met for a fuller understanding of OPs.

## Opportunistic pathogens and a challenge to virulence evolution theory

The study of infectious diseases has become a major focus within evolutionary biology; however, remarkably little attention has been paid to an extremely broad class of pathogens, the opportunists. This oversight stems from the theoretical convenience of treating host–parasite interactions as closed systems in which a single, obligate pathogen specialises on a single host [1,2]. Most pathogens actually fail to meet these assumptions, with many coexisting relatively peacefully with their human host (i.e., they are not obligately pathogenic) or even exploiting an entirely different environment outside of human hosts [3].

Opportunistic pathogens (OPs) are typically characterised in the medical literature as organisms that can become pathogenic following a perturbation to their host (e.g., disease, wound, medication, prior infection, immunodeficiency, and ageing). These opportunists can emerge from among the ranks of normally commensal symbionts (e.g., *Streptococcus pneumoniae* and *Staphylococcus aureus*) or from environmentally acquired microbes (e.g., *Pseudomonas aeruginosa* and *Burkholderia cepacia*). Many more pathogens are recognised as opportunists in the sense that although they regularly cause disease in healthy humans, they are also zoonotic and exploit numerous other hosts (e.g., *Bacillus anthracis* and rabies virus).

We propose a broad and simple definition of OPs: non-obligate and/or non-specialist parasites of a focal host. Thus, if the classic assumptions (obligate parasite and specialist on one host) of virulence evolution theory fail, we have an OP. In Table 1 we outline, with examples, how the combinations of these two conditions give us a classification of OPs into commensal opportunists, environmental opportunists and parasite opportunists (or zoonoses).

Given the failure of the two key assumptions of classical virulence evolution theory (Table 1), what can this body of theory tell us about OPs? For some parasites, the strict failure to meet these assumptions might not be important if the approximation is reasonable in practice, for instance if humans are the major source of parasite transmission (to any host) and the parasite does not routinely enter a commensal stage (e.g., *Salmonella enterica* among humans living in dense and unsanitary conditions). In these cases, standard predictions from virulence evolution theory may still apply, such as a trade-off between transmission and virulence [4,5]. Yet as the biological reality moves further away from these assumptions, we are left only with the prediction that multi-environment opportunists are likely to experience non-optimal virulence in a given host [6,7]. However, ecological and evolutionary theory offers an increasing number of insights into other key features of many opportunists, in particular plasticity and generalism. All human OPs are generalists in the sense that they are able to grow in more than one environment. In addition, many OPs display remarkable phenotypic plasticity, being able to modify phenotypic expression as a function of their changing environmental context.

If we can understand microbial plasticity and generalism, we can understand why opportunistic bugs become pathogens, when they are likely to do this and how we can interfere with their plastic responses to control their virulence in a sustainable manner. In this review, we aim to develop a general and integrative framework for the understanding and management (on ecological and evolutionary time scales) of opportunistic pathogens.

## What is virulence, and why damage your host?

For population biologists, virulence is typically the increase in host mortality resulting from parasite infection [1]. Although this is an explicit and measurable quantity, it ignores many aspects of parasite biology that cause harm without death [6,8–10]. For medical microbiologists, virulence is understood as harm or morbidity to the host, but the focus is on the mechanistic basis of harm, such as identifying virulence determinants or factors (VFs). VFs are typically defined as pathogen components whose loss specifically impairs virulence but not viability (in rich media); classic bacterial examples include toxins, exoenzymes, adhesins, and secretion systems [11].

VFs can be mechanistically complex and therefore are presumably products of natural selection. However, the nature of selection for maintaining and strengthening VFs remains controversial. Levin and Svanborg Edén made an important distinction between direct and coincidental selection for VFs [12]. Under direct selection (by far the most influential model), expression of the VF is correlated with the ability of the parasite to exploit and/or be transmitted from the host; in other words, parasitic VF expression (and consequent costs in terms of host mortality) is rewarded by some benefit. These benefits can either be gained through transmission [1,2] or through within-host growth [13]. This dichotomy, highlighting the importance of multiple scales in disease processes [14], forms the basis of the standard evolutionary view of virulence.

By contrast, purely coincidental selection argues that VF expression is not positively correlated with any measure of parasitic success within the focal host; in other words, there are no benefits in the parasitic context. VFs are then fascinating spandrels [15,16], byproducts of selection for adaptations not related to disease. The mystery of why a VF exists must then be answered elsewhere in the parasite life history, with VF maintenance caused by some benefit in an extra-host habitat or a within-host habitat in which the organism does not cause disease. In this case, can we still make general statements about the dynamics (ecological and evolutionary) of virulence, or are we relegated to case-by-case considerations [12,17]? To begin to answer this challenge, we develop a descriptive model framework to outline how four key selective pressures (coincidental, colonisation, export, and within-host) can combine to shape the evolutionary dynamics of VFs.

## Virulence factor dynamics across multiple environments

**Figure 1:**
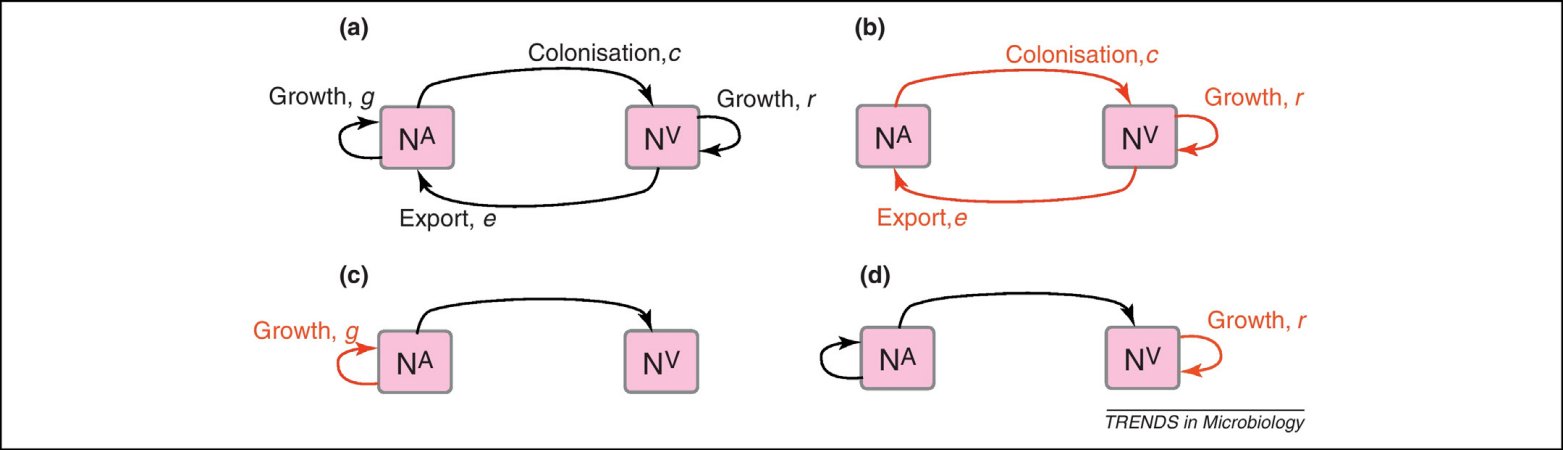
Ecological and evolutionary dynamics of virulence factors across two growth environments. N^A^ and N^V^ represent bacterial densities in the asymptomatic and virulence sites, respectively. Arrows represent demographic processes of growth (*g, r*) and transmission (colonisation *c*, export *e*). Red arrows represent a positive selective impact of VF expression. **(a)** Our basic ecological model (see Box 1 for analysis). **(b)** Epidemiological selection [1,2]; the dominant theoretical paradigm for virulence evolution states that virulence factors yield a net benefit during parasitic exploitation because of positive effects on within-host growth and/or transmission. **(c)** Purely co-incidental selection [12]. **(d)** Purely within-host selection [13].

A characteristic of all opportunistic pathogens is compartmentalisation into environments where they cause disease (e.g., burn wounds for *P. aeurignosa* and the circulatory system for *S. pneumoniae*) and environments where they do not (soil and nasopharynx, respectively). This compartmentalisation can be within a focal host (in particular, either side of mucosal barriers) or between a focal host and another environment (e.g., animal reservoirs vs human hosts). To conceptualise this split, we divide the world of a microbe into two compartments: the virulence compartment V, the sensitive parts of a focal host where microbial VFs result in disease symptoms; and the asymptomatic compartment A, which is everywhere else it can grow (Figure 1a, schema inspired by [18]). In Figure 1b–d, we illustrate how four selective pressures (coincidental, colonisation, export and within-host; the four arrows in Figure 1a) can combine to recover existing theories on the evolution of virulence (Box 1).

The analysis in Box 1 illustrates that if the within-host and transmission (colonisation and export) pressures select against VFs, then there are no countervailing benefits of VFs during host exploitation or transmission (and VFs will be less common in virulence compartments, variation permitting): we are left with purely coincidental virulence (Figure 1c). Pure coincidental virulence implies a positive association with damage but not growth or transmission from V*.* A classic example is found in the soil bacterium *Clostridium botulinum*: botulinum toxin is an extremely potent virulence factor when introduced into humans, but *C. botulinum* itself cannot grow in, let alone be transmitted from, humans [12] (thus, humans are an ecological sink [19]). The simple formulation in Box 1 therefore clarifies how and why some empirical studies may fail to find a selective advantage to VFs in infections (site V). Even without any benefit in site V, selection could favour VF expression, depending on the frequency at which bacteria encounter sites A and V and the relative magnitude of benefits (replication in site A) and costs (growth in site V or movement between A and V). We now use the framework outlined in Figure 1 and Box 1 to discuss the importance of positive and negative correlations between bacterial environments A and V.

## Pre-adaptation

**Figure 2:**
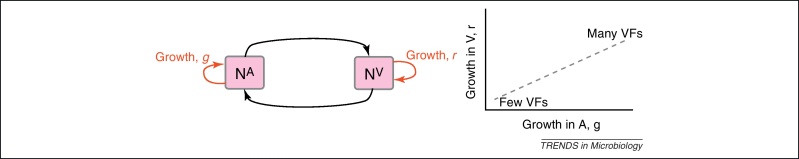
Adaptation to a benign environment A can pre-adapt an opportunistic pathogen for virulent growth in V if there is a significant positive association between the properties of environments A and V (i.e., if fast growth in A, *g*, is correlated with fast growth in V, *r*, or cov(*g,r*) > 0).

Pure coincidental selection as exemplified by *C. botulinum* virulence is, however, an extreme situation: coincidental selection can also coexist with positive within-host and/or transmission selection for VFs. In these cases, the VFs are multi-functional. If we assume that environment A is the primary site of adaptation, then we can conclude that selection in site A generates pre-adaptations for the virulent exploitation of site V (i.e., once in site V, the VF confers some advantage in terms of growth or transmission, but evolution of the VF was driven by selection in site A). If, for example, both coincidental and within-host selection favour VF expression, then there is a positive environmental correlation between growth in environment A and that in environment V (the site of virulence, Figure 2).

An emerging paradigm of VF pre-adaptation driven by environmental correlation is the ability of bacteria to generalise mechanisms for resisting protists for use in other situations. Protists are an important class of bacterial predators across diverse environments (including within host-associated microbiotas), and increasing evidence points to the evolution of resistance to protist predation pre-adapting certain environmental microbes for survival and even proliferation within human macrophages [20–26]. For example, Steinberg and Levin demonstrated that a Shiga toxin VF of *Escherichia coli* O157:H7 increases survival in the presence of grazing protozoa [25]. This result suggests that protozoan predation within ruminants or in the soil may have selected for the VFs that drive pathogenicity and, in particular, export and transmission through Shiga toxin-induced diarrhoea in humans.

Other potential examples of pre-adaptation include selection for capsule carriage (a VF increasing the risk of invasive disease) among pneumococcal strains in the nasopharynx [27]. The most common disease states caused by *S. pneumoniae* are pneumonia, otitis media and sepsis, and these are not contagious conditions and therefore represent a dead end, especially when the result is rapid demise of the host [28]. Rather, transmission occurs from the reservoir of pneumococci residing asymptomatically in the nasopharynx during the organism's commensal state [29]. However, among the more than 92 types of pneumococci expressing structurally distinct capsular polysaccharides, only a few are potentially virulent [30,31]. So why has the pneumococcus evolved or maintained the capacity for virulent, invasive behaviour through the expression of certain thick capsular polysaccharide coats? The results of Lysenko *et al.* demonstrate that capsule selection is driven in the nasopharynx by competitive interactions with another commensal, *Haemophillus influenzae* [27]. While pneumococcal growth is suppressed by *H. influenzae*, the capsule offers a survival advantage by reducing susceptibility to this suppression. These results also present an important reminder that OPs will often face many distinct non-virulent environments (various A, A′, etc.), such as environments with or without a key predator or competitor (here, *H. influenzae*).

Lysenko *et al.* illustrate that growth in a crowded, immunogenic nasopharynx selects for serum resistance, which then pre-adapts *S. pneumoniae* for growth in blood [27]. Similarly, survival against protists (in soil, say) selects for survival against macrophages (in human hosts) [20–26]. Both of these examples highlight that shared or similar environmental challenges can shape the potential for new outbreaks, by building positive correlations between environments (Figure 2).

## Environmental tradeoffs: specialism and plasticity

**Figure 3:**
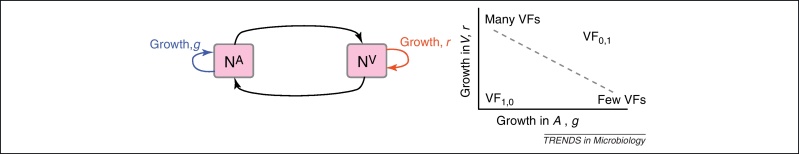
Adaptation to a benign environment A can reduce the capacity for virulent growth in V (and vice versa) if there is a significant negative association (trade-off) between growth rates *g* and *r.* (A negative selective impact of virulence factor expression is denoted by the blue arrow.) Plasticity can decouple the trade-off by expressing the V-specific virulence factor (VF) in V and not in A (VF_0,1_, where the subscripts denote expression in the two environments). However, plasticity also allows a ‘worst of all worlds’ outcome, VF_1,0_, whereby virulence factors are expressed inappropriately (here, only in A).

The examples above describe cases in which selection for VFs may have occurred in a setting outside of infection, but incidentally provides some benefit in terms of transmission or within-host growth (Figure 2). Alternatively, the association between growth in A and V can be negative (Figure 3). How do OPs deal with such environmental trade-offs? A first possibility is that they do not: the focal lineage continues to adapt to its primary environment A, and in certain V environments, bacteria will be unsuccessful. This would be a reasonable strategy if V environments were infrequently encountered and/or unproductive (*C. botulinum* is a candidate here). However, if bacteria frequently encounter environments across which the costs of the trade-off are felt (and if sufficient genetic variation exists), then something is likely to give: evolution in the face of an important trade-off can lead to a loss of the trade-off (if the underlying constraint is weak), specialisation or plasticity.

A common motor of bacterial specialisation is horizontal gene transfer and loss; plasmids and phages shuttle an array of genes conferring local adaptations to heterogeneous environments [32,33], including a strikingly large number of VFs [12,34,35]. The acquisition of VFs via horizontal transfer can render harmless bugs more pathogenic, switching (specializing) or even extending (generalizing) their environmental repertoire. Turner *et al.* posed the question as to whether generalists or specialists would be better able to exploit an entirely novel host type, previously unseen by either line [36]. In other words, which would make the better OP? They illustrated that specialist RNA viruses (evolved under a single host condition) were able to outperform generalists in specific novel host challenges, highlighting the importance of coincidental (or indirect) selection. However, generalists tended to be more consistent across a range of novel challenges, suggesting that consistency is characteristic of generalists. Generalist phenotypes, whether selected directly or indirectly, result from either increased phenotypic constancy across environmental variation or plasticity (phenotypic switching) [37]. For OPs there are many examples of remarkable plasticity that we now discuss.

Plasticity is the ability of an organism to change its phenotype without corresponding changes in genotype. Mechanisms such as altering gene expression can allow an organism to display different phenotypes in different environments [38], and when these responses match the changing environmental requirements (i.e., improve the organism's fitness in that environment) this is called adaptive phenotypic plasticity. Standard theory for virulence evolution has only recently started to incorporate phenotypic plasticity [39,40], but for OPs this phenomenon is of clear importance.

Bacterial VFs are by definition ‘optional extras’ and are often under regulatory control and are not always on, with expression responsive to both physical (e.g., pH and temperature) and social (e.g., density and diversity) environmental dimensions [41–44]. The underlying regulatory machinery is highly complex and variable in extent, with the number of global regulatory sigma factors varying from three in the specialist *Helicobacter pylori* to 24 in the generalist *P. aeruginosa* [45]. This variation in regulatory investment makes sense in the light of plasticity theory: it is only the challenge of frequent exposure to distinct environments that selects for adaptive phenotypic plasticity, in which case the benefits of adaptive plasticity outweigh the likely costs of the machinery necessary to generate such plasticity [46].

Although the direct costs and benefits of a complex regulatory machinery are readily appreciated, there is also potential for indirect costs of making ‘bad decisions’ (Figure 3), as hinted by recent findings for *P. aeruginosa*. On initial colonisation of a mammalian host*, P. aeruginosa* upregulates an array of VFs [41,42]. However, during subsequent evolution in chronically infected cystic fibrosis patients, many of these VFs are subsequently lost, leading to a reduction in the ability to cause acute disease and mortality [47–49]. It has been argued that the loss of secreted VFs may be caused by social interactions favouring ‘cheater’ strains that do not pay the costs of collectively useful VF production [50,51]. However, the continued ability of these strains to persist chronically [48] suggests the possibility that VFs are redundant in the cystic fibrosis lung, and their initial upregulation was a ‘bad decision’ (alternatively, the benefits of VF expression may change through the course of infection as the infection environment develops).

The causes of some aspects of this decision-making have been brought into closer focus for the *P. aeruginosa* toxin pyocyanin, expression of which is driven by exposure to *N*-acetylglucosamine and its polymer peptidoglycan, commonly shed by Gram-positive bacteria [52]. In addition to damaging eukaryotic cells, pyocyanin is a potent antimicrobial, suggesting that *N*-acetylglucosamine-dependent pyocyanin expression is an antimicrobial mechanism in environments rich in competitors [52]; this may then be triggered inappropriately in the cystic fibrosis lung due to human-derived *N*-acetylglucosamine. There are also many well-studied examples of global regulation in quorum-sensing and stress responses (like RpoS in many proteobacteria) that strongly impact virulence [53,54]. The impressive and expanding mechanistic understanding of bacterial plasticity (regulatory control) provides a particularly rich arena for evolutionary investigation, with clear importance for applied questions of bacterial control.

## Managing antibiotic resistance and virulence

Finally, we turn to the implications of opportunism for parasite control. If most human pathogens are largely shaped by selective pressures outside of disease-causing compartments, then why is antibiotic resistance such a clear and growing problem? A major part of the answer is that for many VFs discussed above, antibiotic resistance genes can confer advantages outside of the context of human medical interventions via resistance to bacterially derived antimicrobial compounds. Consistent with this broader functionality, resistance to a range of antibiotics have been found in ancient DNA from 30 000-year-old permafrost sediments [55]. Nevertheless, antibiotic resistance has spread rapidly in many bacteria since the introduction of antibiotics into medical and farming practice [56], indicating that selective pressures are stronger in patients than in nature.

For commensal opportunists, exposure to antibiotics is routine because of their specialisation on human hosts, and therefore the emergence of antibiotic resistance in these species poses little puzzle. By contrast, non-specialists may encounter humans merely as a dynamical ‘sink’ [19], and thus human interactions are unlikely to drive the evolution of antibiotic resistance genes among these populations. However, resistance may pose a significant problem in these lineages because of to a mix of innate resistance properties [57] and shuttling of resistance genes by horizontal gene transfer, particularly during chains of human–human transmission [58].

Interest is now growing in the use of antivirulence drugs that directly target the expression of virulence factors [59–64]. It has been argued that these drugs will limit the evolution of resistance, because they do not kill or halt the growth of their targets [59,60]. How does this claim stand up in the context of OPs? If bacteria only see the drug in V (the virulence site) and the VF is only selected for in A (purely ‘coincidental selection’), then the drugs have potential: the antivirulence drug in this context will enhance a natural tendency towards virulence attenuation within hosts. However, if bacteria see the drug in their ‘non-virulent’ compartment (A) and/or the VF is positively correlated with transmission, then the risks are far greater. A simple implication is that these drugs will hold more long-term promise for the treatment of environmental opportunists because of greater isolation between compartments A and V.

## Concluding remarks

Although there is a broad range of conceptual models for the evolution of virulence (Figure 1, Box 1), formal mathematical treatments have focused overwhelmingly on the most tractable subset, the specialist, obligate parasite [1,2,65]. Here we argue that this bias has hindered effective evolutionary studies of opportunistic pathogens. The admission of multiple growth environments inevitably makes the mathematics more complex (Box 1) [6,66]. More importantly, it also highlights the extent to which biological details matter, with selection on VFs dependent on a complex web of environmental correlations that are only beginning to be picked apart via careful study of bacterial population biology inside and outside of the sites where bacteria cause disease [20–27,52].

Our formal treatment was presented in the context of distinct physical environments (e.g., blood versus mucosa); however, the control of bacterial VF expression in response to contrasting social conditions highlights an even greater complexity and a key theoretical challenge. For instance, many bacteria can discriminate between low- and high-density environments, and even clonal versus polymicrobial conditions, via quorum-sensing mechanisms [67–69] and cues [52]. The development and testing of a novel theory integrating the molecular, ecological and evolutionary dynamics of VFs across complex social and physical environments hold real promise for accelerating our understanding of VFs and their potential as targets for evolutionarily robust antivirulence drugs.

## Figures and Tables

**Table 1:** An ecological classification of pathogens with representative examples

	Obligate parasite	Facultative parasite
Specialist on humans	Current virulence evolution paradigm: *Plasmodium falciparum*, HIV, influenza virus (A, B, C), *Mycobacterium tuberculosis*	Commensal opportunists: *Staphylococcus aureus*, *Enterococcus faecalis*, *Haemophillus influenza*, *Streptococcus pneumoniae*
Non-specialist on humans (generalist)	Parasite opportunists (zoonoses): *Borrelia burgdorferi*, rabies virus, *Salmonella* spp., *Bartonella henselae*	Environmental opportunists: *Pseudomonas aeruginosa*, *Burkholderia cepacia*, *Rhodococcus equi*, *Mycobacterium marinum*, *Vibrio vulnificus*

## Box 1 Ecological and evolutionary dynamics in structured environments

In Figure 1a, we introduce a simple diagrammatic sketch of bacterial population growth in two compartments, one incurring virulence (site V) and the other asymptomatic (site A). To track population dynamics across these two compartments, we define the number of microbes of strain *i* in each compartment as *N*^A^_*i*_ and *N*^V^_*i*_. Microbes replicate in the asymptomatic site, A, at a net per capita rate *g* and leave this site to colonise the symptomatic (virulence) site, V, at rate *c*. Within this symptomatic site of the host, microbes grow at rate *r* and are released back into the asymptomatic site (e.g., through bacterial shedding) at rate *e*. Each microbial strain may have unique rates for these processes, so each rate parameter is subscripted by *i*. This gives rise to the dynamic equations below for tracking the strain-specific population dynamics of bacteria in each compartment:

$$\frac{\text{d}N_{i}^{\text{A}}}{\text{d}t}=g_{i}N_{i}^{\text{A}}+e_{i}N_{i}^{\text{V}}\text{,}$$

$$\frac{\text{d}N_{i}^{\text{V}}}{\text{d}t}=r_{i}N_{i}^{\text{V}}+c_{i}N_{i}^{\text{A}}\text{.}$$

To address evolutionary questions of change in VF investment, we allow each of these vital rates (*g*, *c*, *r*, *e*) to be functions of VF expression, *v*. Here, the trait *v* could be the number of VFs expressed or the level of expression of a given VF. We can now use the epidemiological Price equation approach to study the forces acting on *v* in sites A and B [70]. Following the maths in Day and Gandon [70] (but ignoring mutation between types), the rate of change in the average level of VF expression, $\bar{v}$, in each site is given by:

$$\frac{\text{d}{\bar{v}}^{\text{A}}}{\text{d}t}={\text{cov}}_{\text{A}}\left(v_{i},g_{i}\right)+\frac{N^{\text{V}}}{N^{\text{A}}}\left({\text{cov}}_{\text{V}}\left(v_{i},e_{i}\right)+\bar{e}\left({\bar{v}}^{\text{V}}-{\bar{v}}^{\text{A}}\right)\right)\text{,}$$

$$\frac{\text{d}{\bar{v}}^{\text{V}}}{\text{d}t}={\text{cov}}_{\text{V}}\left(v_{i},r_{i}\right)+\frac{N^{\text{A}}}{N^{\text{V}}}\left({\text{cov}}_{\text{A}}\left(v_{i},c_{i}\right)+\bar{c}\left({\bar{v}}^{\text{A}}-{\bar{v}}^{\text{V}}\right)\right)\text{.}$$

In both equations, the first terms represent the effects of direct selection on *v* in each site. In other words, natural selection will favour an increase in the average value of *v* in site A (or V) if bacteria with higher VF expression also have higher growth rates in site A (or V). The second and third terms represent changes in the average *v* as a result of movement between sites A and V, weighted by the population sizes in the two sites. For example, the average value of *v* in site A will tend to increase if bacteria with higher VF expression also have higher rates of export from site V to A [i.e., cov_V_(*v*_*i*_,*e*_*i*_) is positive]. The average value of *v* could also increase by export if the average *v* in site V is higher than in site A. The average value of *v* in site V could similarly change through colonisation from A to V. In the main text, the importance of the four highlighted covariance terms is discussed, along with their relationship to existing theories for the evolution of virulence (Figure 1).
